# Molecular Signatures of Cancer Stemness Characterize the Correlations with Prognosis and Immune Landscape and Predict Risk Stratification in Pheochromocytomas and Paragangliomas

**DOI:** 10.3390/bioengineering12030219

**Published:** 2025-02-21

**Authors:** Lei Li, Shuangyu Liu, Zeqi Guo, Yueming Tang, Yue Zhang, Ling Qiu, Yue Li

**Affiliations:** 1 Department of Laboratory Medicine, Peking Union Medical College Hospital, Peking Union Medical College & Chinese Academy of Medical Science, Beijing 100730, China; lilei30@pumch.cn (L.L.); tangyuem1@126.com (Y.T.); 2 Department of Clinical Laboratories, The Second Affiliated Hospital of Xi’an Jiaotong University, Xi’an 710004, China; liushuangyuyu@126.com (S.L.); sxycgzq@126.com (Z.G.); 18317009638@163.com (Y.Z.); 3 State Key Laboratory of Complex Severe and Rare Diseases, Peking Union Medical College Hospital, Peking Union Medical College & Chinese Academy of Medical Science, Beijing 100730, China

**Keywords:** pheochromocytoma and paragangliomas, tumor stemness, tumor microenvironment, prognostic model

## Abstract

Background: Pheochromocytoma and paragangliomas (PPGLs) caused refractory hypertension in clinics. The sustained risk of local or metastatic recurrences or new tumor development prompted more research on diagnosis, prognosis prediction, and immunotherapy. Method: The tumor stemness is closely related to the heterogeneous growth of tumor, metastasis, and drug-resistance, and mRNA expression-based stemness indices (mRNAsi) could reflect tumor stemness. This was calculated based on OCLR machine learning algorithm and PPGLs patients’ TCGA RNAseq data. The relationship between clinical, molecular, and tumor microenvironment (TME) features and tumor stemness was analyzed through the hub genes that best captured the stem cell characteristics of PPGLs using weighted gene co-expression network analysis (WGCNA), Cox, and LASSO regression analysis. Results: Our study found that metastatic PPGLs had higher mRNAsi scores, suggesting the degree of tumor stemness could affect metastasis and progression. *HRAS*, *CSDE1*, *NF1*, *RET*, and *VHL*-mutant subtypes displayed significant difference in stemness expression. Patients were divided into stemness high-score and low-score subtypes. High-score PPGLs displayed the more unfavorable prognosis compared with low-score, associated with their immune-suppressive features, manifested as low macrophages M1 infiltration and downregulated expression of immune checkpoints. Furthermore, from the viewpoint of stemness features, we established a reliable prognostic for PPGLs, which has the highest AUC value (0.908) in the field so far. And this could stratify PPGLs patients into high-risk and low-risk subtypes, showing the significant differences in prognosis, underlying mechanisms correlated with specific molecular alterations, biological processes activation, and TME. Notably, high immune infiltration and tumor neoantigen in low-risk patients and further resulted in more responsive to immunotherapy. Conclusion: We indicated that tumor stemness could act as the potential biomarker for metastasis or prognosis of PPGLs, and integrated multi-data sources, analyzed valuable stemness-related genes, developed and verified a novel stemness scoring system to predict prognosis and guide the choice of treatment strategies.

## 1. Introduction 

Pheochromocytomas and paragangliomas (PPGLs) are rare clinical tumors with a prevalence of 500 to 1600 cases per year in the general population. They have a wide range of symptoms and indications, ranging from asymptomatic disease to non-specific symptoms, leading to refractory hypertension and catastrophic hypertensive crisis [1]. Pheochromocytomas are tumors that develop from catecholamine-secreting chromaffin cells in the adrenal medulla, whereas paragangliomas are tumors that arise from extra-adrenal sympathetic or parasympathetic paraganglia [2]. Although the two are located in different anatomical locations, their histological manifestations are highly similar due to their common origin in neural crest cells. Surgical excision could help control the large proportion of tumors. However, patients display a wide range of different clinical courses; nearly 15–20% of patients metastasize to the liver, bones, and lungs, which are distant from the primary tumor. There remains a sustained risk of local or metastatic recurrences or the development of a new tumor [3,4]. In the past decade, immunotherapy has obtained promising results in several types of solid cancers by applying novel strategies that strengthen the natural immune antitumor military. However, immunotherapy for PPGLs is in the early stages of development; we must better understand the mechanisms related to immune system activation.

The cancer stem cells (CSCs) hypothesis postulates that a subpopulation of tumor cells is capable of self-renewal and is responsible for long-term tumor maintenance. Furthermore, numerous studies have demonstrated a connection between the biological properties of tumor stem cells and resistance to chemotherapy and radiation therapy, tumorigenesis, metastasis, tumor self-replication ability, tumor metabolic reprogramming, and tumor immune microenvironment remodeling, suggesting that it could be a promising therapeutic target. Recently, a study focusing on non-Hodgkin’s lymphoma demonstrated that *CD47* (a tumor stemness-associated gene) is highly upregulated in the tumor cells of some patients, and is resistant to monoclonal antibody (rituximab) therapy. These tumor cells provide a “do not eat me” signal to defend against being phagocytosed by macrophages. Additionally, tumor stemness is associated with the poor prognosis of some cancers, such as soft tissue sarcomas [5], breast [6], and ovarian cancers [7]. However, few studies focus on the role and impact of CSCs in PPGLs.

In this study, we aim to analyze the CSCs’ characteristics in PPGLs patients from The Cancer Genome Atlas (TCGA) and explore the relationship between cell stemness and tumor immunity. We may develop a stemness scoring system that can be applied to predict prognosis and patient responsiveness to immunotherapy after identifying factors highly correlated with tumor stemness. Additionally, the newly identified predictors of response may help determine the subgroup of patients most likely to respond favorably to this therapy. Finally, new trials are underway, promising to reveal further information on a therapy that marks a major advancement in cancer.

## 2. Materials and Methods

### 2.1. Data Collection and Processing

A total of 178 PPGLs patients were analyzed in this study. The mRNA expressions and follow-up information were obtained from the TCGA website (https://portal.gdc.cancer.gov/) (accessed on 20 June 2023). Gene mutation information (somatic or germline mutation) and molecular subtypes (TCGA subtype and miRNA cluster) were collected from the Supplementary Materials in Fishbein et al.’s study [8]. Malta et al. [9] developed a machine learning algorithm named “OCLR” to define stem cell features based on the stem cell data (stemness associate gene expression, methylation, and mutation). The two dependent stemness indices (mDNAsi and mRNAsi) for TCGA-PPGLs patients were obtained from their literature. The data used in this article was obtained from publicly available databases.

### 2.2. The Clinical and Molecular Features Associated with the Stemness Index (mRNAsi)

Since gene expression data are more accessible than epigenetic data, mRNAsi was chosen to explore their association with phenotypic characteristics. The correlation between clinical features and mRNAsi was constructed using the Beeswarm R package (https://www.rdocumentation.org/packages/beeswarm/versions/0.2.3/, accessed on 25 June 2023), involving gender, age at beginning pathologic symptoms, vital Status, pheochromocytoma or paraganglioma, tumor location, and metastatic disease. The correlation between mutation status or molecular subtypes and mRNAsi was also constructed using the Beeswarm R package 0.2.3.

### 2.3. Associations Between the Stemness Index and PPGLs Immune Signatures

Based on the gene expression profiles of PPGLs samples, the single-sample Gene Set Enrichment Analysis (ssGSEA) method was used to quantify the enrichment levels of tumor-infiltrating immune cells (TIICs) using R package GSEABase. This provides a set of gene signatures for a 28 human tumor microenvironment (TME) immune cell fractions, such as activated T cells, regulatory T cells, and natural killer (NK) T cells. The PPGLs samples were further divided into two stable clusters (high- and low-immunity clusters) according to the scores of ssGSEA using the unsupervised clustering methods of the ConsensuClusterPlus R package. To better comprehend the connection between the amount of immune invading cells and the mRNAsi, we evaluated the correlation between the mRNAsi and immune score, stromal score, and ESTIMATE score, respectively.

The immune score and stromal score representing immune and invasion of stromal cells at the tumor site, respectively, and the ESTIMATE score representing tumor purity, were calculated using the ESTIMATE R package (v3.6.3, https://www.r-project.org/, accessed on 25 June 2023). The relative proportion of 22 immune cells in PPGLs patients was quantified using the CIBERSORT algorithm. The variations in these three immunological correlation scores, the mRNAsi, and immune-related cell types between the high- and low-immunity clusters were compared. The association between the three immunological correlation scores, immune-related cell types, and mRNAsi was estimated using Pearson correlation analysis.

The median value of mRNAsi was chosen as the boundary to divide TCGA-PPGLs patients into the high- and the low-mRNAsi groups. The expressive heterogeneity of immunological checkpoints between the high- and low-mRNAsi groups was determined using unpaired Student *t*-tests.

### 2.4. Functional Enrichment Analysis

The DEGs were determined with fold changes > 1.5 and a statistically significant corresponding *p* < 0.05 using the limma R program. The Gene Ontology (GO)/Kyoto Encyclopedia of Genes and Genomes (KEGG) enrichment analyses were conducted on these DEGs to reveal their potential function. The GOplot R package was used for the z-score calculation (z-score > 0, referring to positive regulation; z-score < 0, referring to negative regulation; a larger absolute value reflects a relatively greater difference in the number of highly and lowly expressed elements, indicating a potentially higher degree of regulation). The representative pathways enriched in molecular function (MF), cellular component (CC), and biological processes (BP) were displayed in chord diagrams in order of absolute Z value, with an adjusted *p*-value < 0.05.

### 2.5. Identification of mRNAsi-Related DEGs and Candidate Target Drugs

The co-expressed gene modules that group similar gene expression patterns were identified via WGCNA co-expression network analysis. The following are the primary stages that comprise the analysis process: (1) obtained the optimal soft threshold power β value through the pick-Soft-Threshold algorithm; (2) the β value was selected to be used in the formation of the proximity matrix, (3) which was subsequently converted into the topological overlap matrix (TOM); and (4) a hierarchical clustering structure was created to incorporate the nearby gene modules.

Correlation analysis was employed to screen the most significant modules associated with stemness levels (mRNAsi and EREG-mRNAsi indices). Following the determination of the crucial stemness-related module, all of the genes contained in the module were utilized to search for possible target drugs for PPGLs using the signature matching methods (XSum algorithm) based on the CMap dataset (CMap Build 2: 3 cell lines, 1309 compounds) [10]. Subsequently, the XSum score ranging from −1 to +1, representing the pairwise similarity metrics between the expression patterns of cells treated with drugs and disease signature, was calculated. Briefly, a drug with XSum scores between −1 and 0 suggested its stronger reversal potency and implementation potential.

### 2.6. Construction of Risk Score Model Based on mRNAsi-Related DEGs

The Kaplan–Meier (K-M) survival analysis was utilized to eliminate the mRNAsi-associated prognostic genes. Furthermore, LASSO regression analysis was conducted to construct the best gene signature derived from the mRNAsi-related prognostic genes using the glmnet R package. The mRNAsi-associated prognostic risk model was created with the following formula: risk score = Expression mRNAi × Coefficient mRNAi, where i represented the sequence of mRNAsi-relevant genes.

The TCGA-PPGLs cohort’s risk scores for each specimen were estimated in line with the model, and the median risk score was chosen as the split point to classify patients into high- and low-risk subgroups. The impact of the risk characteristics on the prognosis of PPGLs in the high- and low-risk subgroups were assessed using K-M curves. Next, receiver operating characteristics (ROC) curves and principal component (PCA) evaluations were used to assess prediction accuracy. A multivariate logistic regression analysis was utilized to ascertain if the risk characteristic could independently reflect the clinical outcome in PPGLs patients.

The relative quantity of the immune cells was calculated in TCGA-PPGLs samples using CIBERSORT [11], EPIC [12], xCell [13], quantTIseq [14], MCP-counter [15], and TIME algorithms [16]. The ssGSEA algorithm was employed to estimate the tumor infiltration fraction and the activities of all steps in the anti-cancer immunity cycle for TCGA-PPGLs samples based on the TIP database (http://biocc.hrbmu.edu.cn/TIP/, accessed on 30 June 2023) [17]. The TIDE algorithm [18] was used to determine the response of the TCGA-PPGLs samples to immunotherapy, allowing the patients to be categorized as non-responders or responders. The diverse immune signatures were compared in high- and low-risk populations, including tumor-infiltrating lymphocyte infiltration, anti-cancer immune activities, several immune checkpoints expression, immune scores, stromal scores, ESTIMATE scores, and immunotherapy response.

The association between immune cells and risk score was assessed using the R package Corrplot based on the data from the immune infiltration obtained through the CIBERSORT algorithm.

It is difficult to discover more PPGLs datasets with prognostic or follow-up data for immunotherapy. The immunotherapy dataset IMvigor210 [19] was chosen as the independent external validation cohort. The patients were divided into high- vs. low-risk groups based on the median value of all risk scores. The prognostic value of the mRNAsi-associated signature was validated using the K-M method. The response for immunotherapy based on the IMvigor210 cohort included complete response (CR), partial response (PR), stable disease (SD), and progressive disease (PD). Immunotherapy responses were evaluated between high- and low-risk subgroups. Meanwhile, we analyzed the differences in genomic mutation landscape and heterogeneity between high- and low-risk subgroups, including tumor mutational burden (TMB), mutant-allele tumor heterogeneity (MATH), microsatellite instability (MSI), tumor neoantigen, homologous recombination deficiency (HRD), and loss of heterozygosity (LOH). Tumor heterogeneity played important roles in the progression, invasion, and drug sensitivity of tumor cells.

### 2.7. Statistical Analysis

Student’s *t*- or Wilcoxon Rank-Sum tests were employed to compare continuous parameters between groups. Spearman’s correlation analyses were used to estimate the relevance between the two parameters of interest. A *p* value less than 0.05 was deemed statistically significant. All the aforementioned were achieved by various R packages implanted in the R Foundation (v3.6.3, https://www.r-project.org/ accessed on 30 June 2023).

## 3. Results

### 3.1. Correlation Between mRNAsi and PPGLs Molecular Mutation

**Figure 1:**
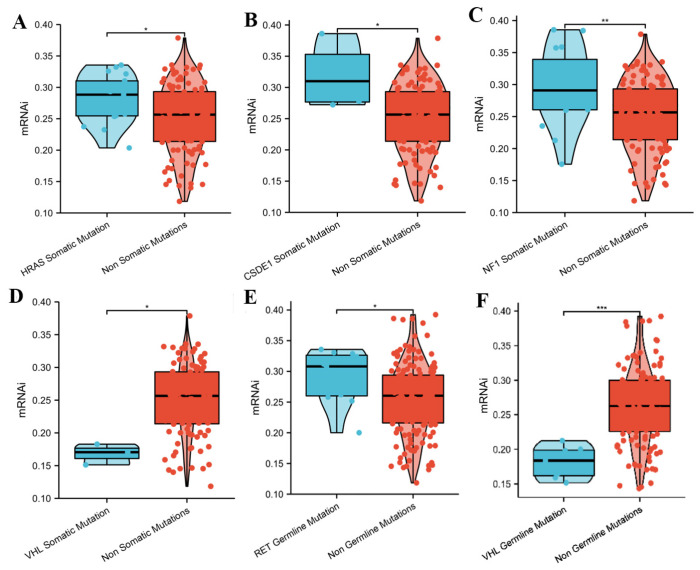
mRNAsi values associated with the mutation status in known PPGLs-susceptibility genes. Comparison of mRNAsi in patients with (**A**–**D**) somatic or (**E**,**F**) germline mutation and wildtype patients. (* *p* < 0.05, ** *p* < 0.01; *** *p* < 0.001).

We examined mRNAsi differences in distinct groups after classifying patients into different groups according to their molecular and clinical state. Figure 1A–C illustrates that the patients with *HRAS*, *CSDE1*, or *NF1* somatic mutation have higher mRNAsi than wild-type patients. We observed that high mRNAsi was associated with *RET* germline mutation when combined with germline mutation data (Figure 1E). However, the stemness index was significantly lower in *VHL*-somatic or germline mutant samples than in *VHL*-wild-type samples (Figure 1D,F). Consistent with earlier studies, the TCGA expression subtypes displayed distinctive mutational profiles; germline and somatic mutations in *VHL* were particular to the pseudohypoxia subtype.

### 3.2. Correlation Between mRNAsi and PPGLs Clinical Features

**Figure 2:**
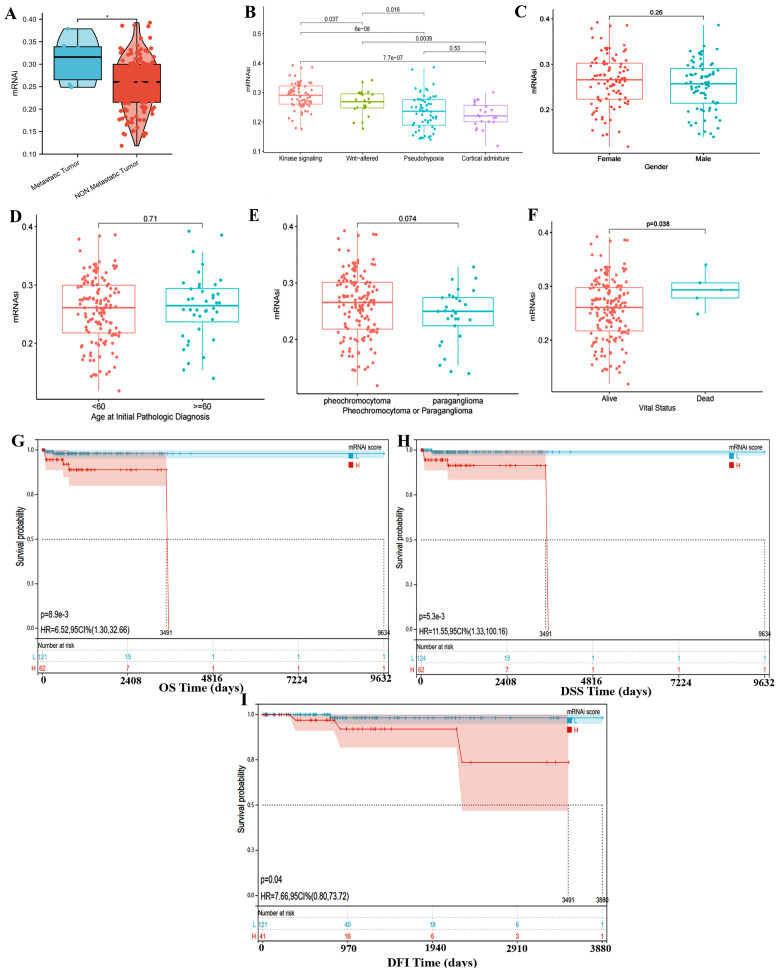
mRNAsi values associated with clinical prognosis and features. Comparison of mRNAsi in (**A**) metastasis occurences, in different (**B**) TCGA-expression subtypes, and (**C**–**F**) clinical characters (gender, age, tumor location, and vital status). (**G**–**I**) Survival and prognosis curve of PGGLs. * *p* < 0.05.

Regarding the clinical features, numerous retrospective studies suggest that patients with *SDHB* mutations are more susceptible to experiencing metastatic disease [20,21]. So, we investigated the connection between mRNAsi and the likelihood of metastasis in patients lacking *SDHB* mutations. Figure 2A shows that the metastatic patients had higher mRNAsi, suggesting that the mRNAsi index may influence the PPGLs malignant process. We also found that the pseudohypoxia subtype showed lower mRNAsi scores than the kinase signaling and the Wnt-altered subtypes (Figure 2B).

Additionally, we discovered that the mRNAsi values were significantly related to clinical outcomes. Figure 2C–F demonstrate that the dead patients have higher mRNAsi scores than alive ones, while patients with different ages, gender, and tumor location of PPGLs did not indicate significant differences in mRNAsi levels. This indicates that miRNAsi may only influence the prognosis of PPGLs, but this cannot be considered as clinically significant evidence due to limited data on death cases. Therefore, the survival analysis was performed, and the results further indicated the K-M curve (Figure 2G–I) shows significant difference in overall survival (OS), disease specific survival (DSS), and disease free interval (DFI) between the two groups with high and low mRNAsi expression in the cases we studied. The high-mRNAsi cluster tended to show a relatively poorer survival outcome, with the shorter OS/DSS or shorter disease-free survival period than low-mRNAsi subtypes(OS, HR = 6.52, 95% CI 1.30–32.66; DSS, HR = 11.55, 95% CI = 1.33–100.16).

### 3.3. Correlation Between mRNAsi and PPGLs Immune Signatures

**Figure 3:**
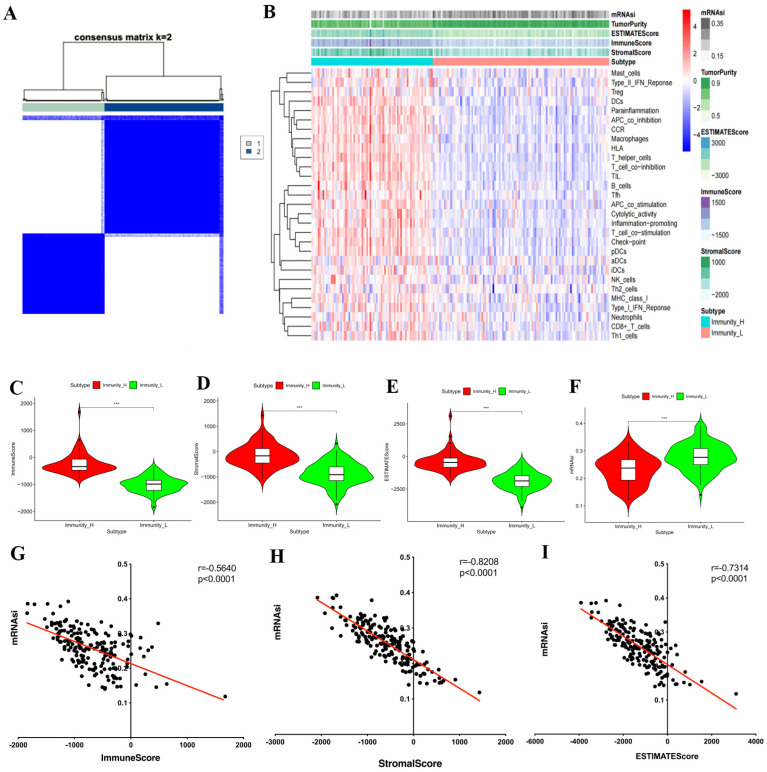
mRNAsi values associated with the immune features of PPGLs. (**A**) Consensus clustering algorithm was employed to obtain stable immune subtypes based on the overall immune activity of PPGLs; (**B**) the overview of differences in 29 immune signatures, immune score, stromal score, ESTIMATE score, and mRNAsi value between the two immune subtypes; violin plots of the (**C**) immune score, (**D**) stromal score, (**E**) ESTIMATE score, (**F**) mRNAsi value of PPGLs patients classified by PPGLs immune subtypes; mRNAsi value was significantly negatively associated with the (**G**) immune score, (**H**) stromal score, (**I**) ESTIMATE score. *** *p* < 0.001.

The ssGSEA analysis was employed to evaluate the TME of the TCGA-PPGLs cohort. Unsupervised clustering was utilized to divide TCGA-PPGLs patients into immunity-H and immunity-L groups (Figure 3A). The high-immunity subgroup was characterized by relatively higher average immune, stromal, ESTIMATE, and mRNAsi scores than the low-immunity subgroup (Figure 3B–F). Meanwhile, we found that the mRNAsi score had a negative association with immune, stromal, and ESTIMATE scores (Figure 3G–I), suggesting that immunosuppression in PPGLs has a significant correlation with the higher degree of stemness.

**Figure 4:**
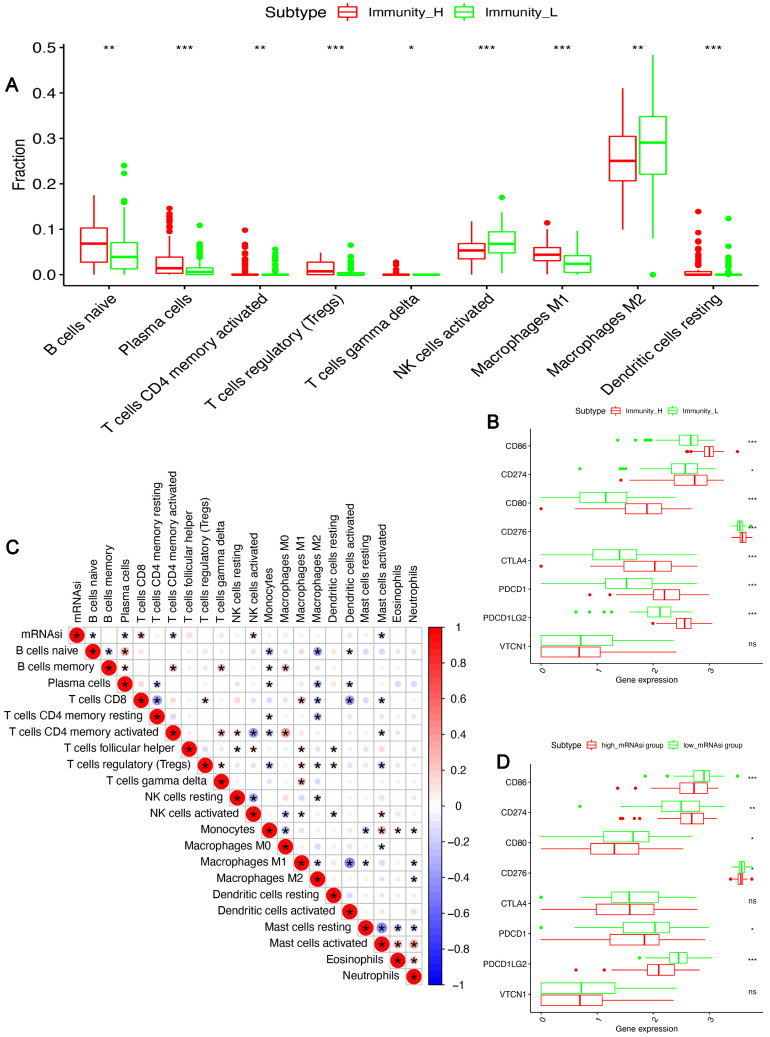
The correlation Between mRNAsi and immune cell infiltration/checkpoint expression. The differences in (**A**) immune cells infiltration and (**B**) immune checkpoint expression levels between the two immune subtypes; (**C**) the correlation between mRNAsi value and immune cell subgroup distribution; (**D**) The differences in immune checkpoint expression levels among the high mRNAsi group vs. the low mRNAsi group. (* *p* < 0.05, ** *p* < 0.01; *** *p* < 0.001, ns, no statistical difference).

Additionally, we compared the TME infiltrating fractions between the two subgroups using CIBERSORT analysis. Figure 4A illustrates that immunity-H has higher anti-tumor TME components like activated memory CD4+ T cells, macrophages M1 cells, and dendritic cells. The correlation analysis revealed a relationship between weak CD4+ T and mast cells and mRNAsi abundance (Figure 4C). We further observed elevated expressions of several immune checkpoints in the high immune infiltration group, such as *CD86*, *CD274*, *CD80*, *CD276*, *CTLA4*, *PDCD1*, and *PDCD1LG2*. However, this trend was reversed in the high mRNAsi group (Figure 4B–D). These findings imply that the tumor stemness index is inversely correlated with TME infiltration. The high mRNAsi group may be linked to poor immune checkpoint treatment efficacy.

### 3.4. Identification of Differentially Expressed Genes and Functional Enrichment Analyses

**Figure 5:**
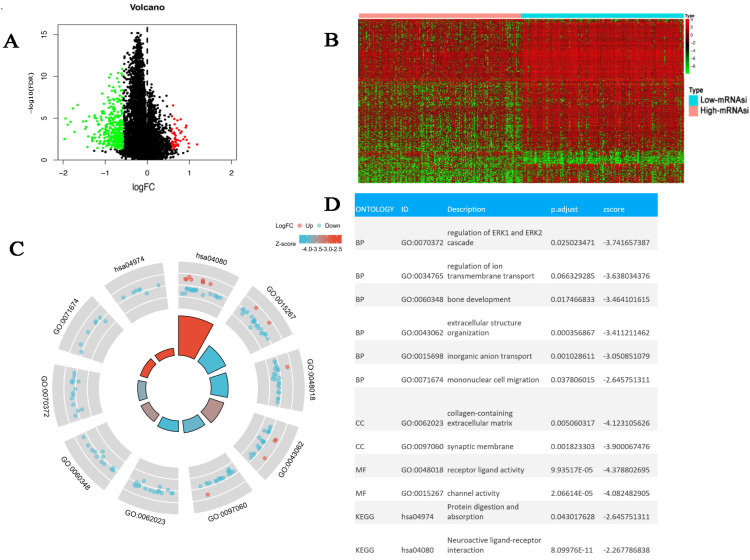
Identification of Differentially Expressed Genes and Functional Enrichment. The (**A**) volcano plot and (**B**) heatmap displayed the expression levels of DEG; (**C**,**D**) GO and KEGG enrichment analysis of DEGs.

A total of 397 DEGs were identified between the high- and the low-mRNAsi groups (Figure 5A,B). We performed the GO/KEGG analysis to investigate the latent biological behavior and the related pathways involved in these DEGs. Figure 5C displays that 397 DEGs mainly enriched several classic tumor signaling pathways, including ERK1 and ERK2 cascade regulation and extracellular structure organization. Protein digestion and absorption, inorganic anion transport, and mononuclear cell migration are active pathways during tumorigenesis and pathways related to immune response regulation. This could explain why the high mRNAsi group had lower immune activity and a higher malignant potential than the low mRNAsi group.

### 3.5. Construction of the mRNAsi-Related Prognostic Model for PPGLs

**Figure 6:**
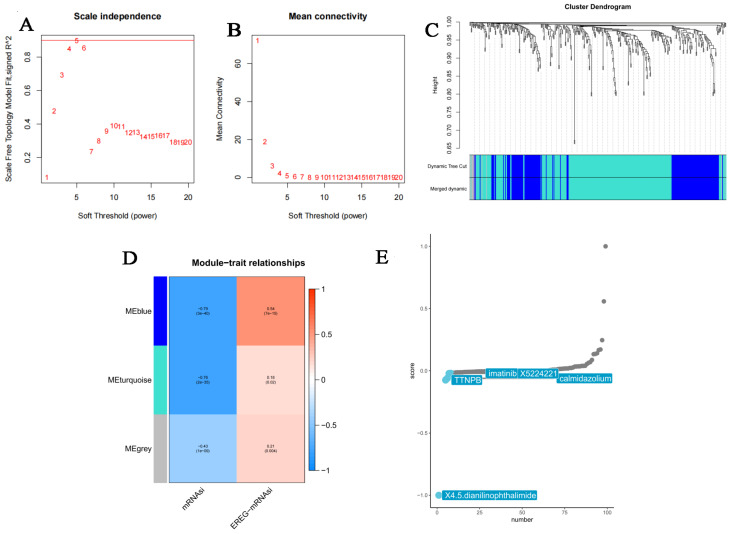
Obtaining the stemness-related gene modules through the WGCNA. (**A**–**C**) selection of the suitable soft-thresholding power to identify weighted gene co-expression modules; (**D**) Heatmap showing the correlation between mRNAsi or EREG-mRNAsi and module eigengenes; (**E**) identification of the potential compounds through CMap analysis.

As previously described, we employed WGCNA to identify the key gene modules of highly correlated DEGs that correlated to mRNAsi and EREG-mRNAsi traits. When soft threshold β = 4, R^2^ > 0.9, this allowed the construction of a scale-free co-expression network (Figure 6A,B). Moreover, DEGs with comparable expression patterns were merged into the same module (Figure 6C). Ultimately, three cohesive gene modules were yielded (Figure 6D). The blue module was the module most relevant to both mRNAsi indices (cor = −0.79; *p* < 0.001) and EREG-mRNAsi indices (cor = 0.54; *p* < 0.001). Then, 141 mRNAsi-related DEGs from the blue module were employed for querying CMap. Five agents (X4.5. dianilinophthalimide, TTBPB, imatinib, X5224221, calmidazolium) were then considered to have anti-stemness potency (XSum score < 0) (Figure 6E).

The K-M survival analysis was utilized to estimate the prognostic value of 141 mRNAsi-related DEGs in PPGLs. Thirty-three genes connected to PPGLs patient’s OS were identified using *p* < 0.05 as the cutoff (Supplementary Table S1).

**Figure 7:**
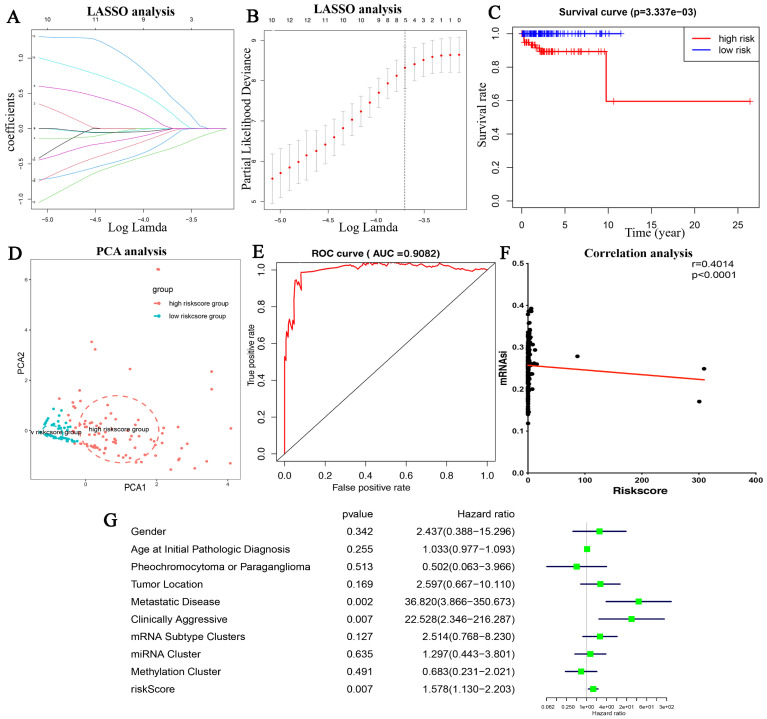
Construction of the mRNAsi-related prognostic model based on the prognostic stemness-related genes. (**A**,**B**) LASSO analysis; (**C**) K-M analysis of risk model; (**D**) PCA analysis; and (**E**) ROC curve of the risk model with significant prognostic prediction value analyzed based on all TCGA-PPGLs patients. (**F**) correlation between mRNAsi values and risk scores (**G**) univariate Cox analysis.

The LASSO algorithm was applied to the 33 prognostic-related genes, and six genes were identified to develop the mRNAsi-risk signature (Figure 7A,B). The six genes were *CCL26*, *GPR148*, *FAM3B*, *GATA4*, *C1orf94*, and *CRYBB3*. Each PPGLs patient risk score was determined as follows:

*CCL26* expression × (0.351137846036265) + *GPR148* expression × (0.601409843037207) + *FAM3B* expression × (−0.746029637361062) + *GATA4* expression × (−1.04632515461906) + *C1orf94* expression × (1.0041025435075) + *CRYBB3* expression × (−0.446009476652402).

The risk score for each TCGA PPGLs patient can be generated by applying this risk model. We observed that the mRNAsi score was positively associated with the risk score (Figure 7F). All TCGA-PPGLs patients were divided into two clusters using the median risk score. Figure 7C displays that the group with high levels of risk score had a greater number of fatalities than the low mRNAsi risk group. The PCA analysis (Figure 7D) showed that the high- and low-risk patients had clinical outcomes about OS. The model’s projected accuracy was assessed using the site under the ROC curve, and it showed outstanding prognostic efficacy (AUC = 0.9082) (Figure 7E). Additionally, univariate Cox analysis demonstrated that clinical aggressive status and risk scores were closely related to PPGLs prognosis (Figure 7G). The prognostic performance of this cancer stemness-based prediction model suggested that it can achieve satisfactory predictive accuracy in survival analysis for PPGLs patients.

### 3.6. The mRNAsi Risk Group Displayed Distinctive TIME and Immunogenomic Profiles

**Figure 8:**
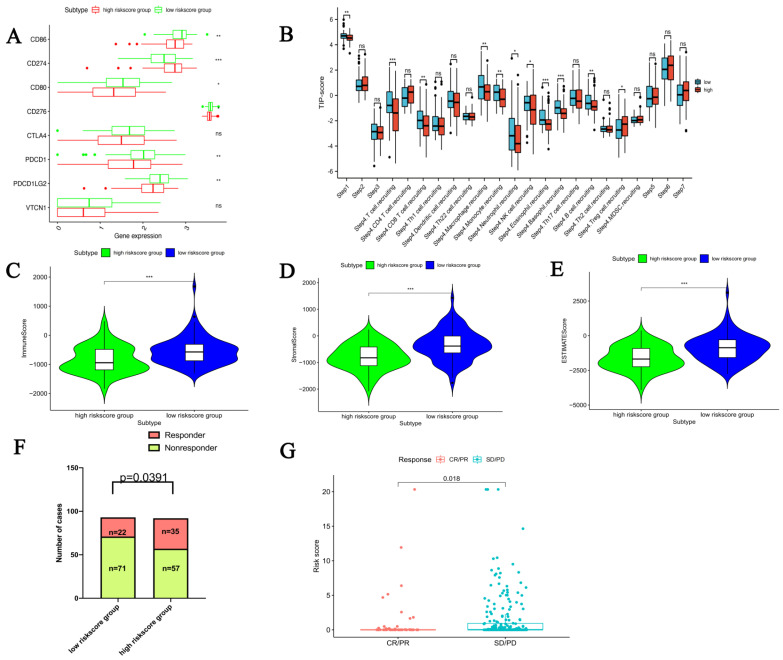
The mRNAsi Risk subtypes displayed distinctive TIME and immunogenomic profiles. The differences in (**A**) immune checkpoint expression levels and (**B**) anti-tumor immunity between the two mRNAsi risk subtypes; comparison of (**C**) immune score, (**D**) stromal score, (**E**) ESTIMATE score among the two mRNAsi risk subtypes; comparison of the therapeutic efficacy between high-risk and low-risk subtypes in (**F**) TCGA-PPGLs cohort and (**G**) IMvigor210 cohort. (* *p* < 0.05, ** *p* < 0.01; *** *p* < 0.001, ns, no statistical difference).

We performed the CIBERSORT, EPIC, xCell, quantTIseq, MCP-counter, and TIME algorithms to assess the relative abundance of immune-associated cell types based on the expression patterns of PPGLs patients (Figure S1A–G). We detected that at least five algorithms elucidate the macrophages, especially the M1 macrophages, were more abundant in low-risk patients (Figure S1B–G). In addition, low mRNAsi risk was associated with high immune checkpoint expressions, such as *PDCD1*, *CD276*, *CD80*, *PDCD1LG2*, and *CD86* (Figure 8A). Meanwhile, we observed more immune-related functions like tumor killing, NK cells, and neutrophils in low-risk patients. Patients with low tumor risk exhibited higher anti-tumor immunity than high tumor risk (Figure 8B). Correspondingly, the immune, stromal, and ESTIMATE scores were negatively related to the risk score (Figure 8C–E). Furthermore, the analysis revealed that PPGLs patients with a low mRNAsi risk were more responsive to immunotherapy (Figure 8F). To further verify the prognostic value of the mRNAsi risk model for clinical outcomes and immunotherapy sensitivity, an independent external validation cohort verified the risk score model based on six mRNAsi-related DEGs. In the IMvigor210 cohort, we used the same method to assign 348 patients to low-risk groups (N = 174) or high-risk groups (N = 174). As expected, patients in the low-risk group had a higher CR/PR rate (Figure 8G).

**Figure 9:**
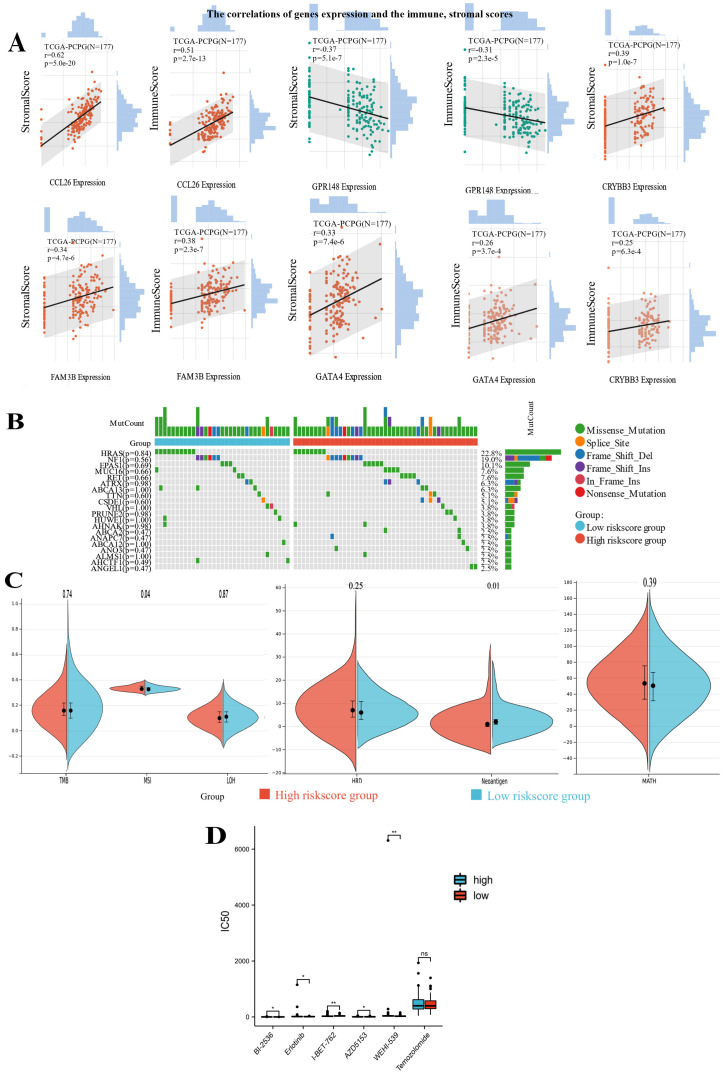
The immune landscape, gene mutation landscape and therapeutic Drug Sensitivities in different mRNAsi risk subtypes. (**A**) The correlations of *CCL26*, *GPR148*, *FAM3B*, *GATA4*, and *CRYBB3* expression and the immune, stromal scores; The differences in genomic mutation landscape (**B**) and genomic heterogeneity biomarkers (**C**) between the two mRNAsi risk subtypes. (**D**) Comparison of the estimated IC50 values of Erlotinib, I-BET-762, AZD5153, and WEHI-539 between the high- and low mRNAsi groups. (* *p* < 0.05, ** *p* < 0.01, ns, no statistical difference).

Meanwhile, we also could observe that *CCL26*, *GPR148*, *FAM3B*, *GATA4,* and *CRYBB3* expression were significantly associated with the immune and stromal scores (Figure 9A). Genomic heterogeneity characteristics, for instance, TMB, MSI, and tumor neoantigen, were important predictors for tumor immunotherapy response within the tumor microenvironment. In our study, although there was no significant difference in the genomic mutation landscape between the high- and low-risk subtypes, we noticed that the low-risk PPGLs patients showed higher tumor neoantigens (Figure 9B,C). Therefore, the favorable prognosis of low-risk patients was found to correlate with the immune microenvironment (immune cell infiltration), high immune checkpoint expressions and tumor neoantigen.

### 3.7. The mRNAsi Risk Group Displayed Distinctive Therapeutic Drug Sensitivities

Temozolomide (TMZ) was the recommended chemotherapy drug for PPGLs postoperatively, however, we found that there was no significant difference in TMZ response between the two groups. Subsequently, we determine whether distinct mRNAsi risk groups exhibited different susceptibility to other therapeutic drugs. Patients with a high mRNAsi risk had higher estimated IC50 values for Erlotinib, I-BET-762, AZD5153, and WEHI-539 (Figure 9D).

## 4. Discussion

Numerous types of research demonstrated that CSCs were related to the self-renewal and long-term tumor maintenance of the subpopulation of tumors [22,23]. Additionally, it has been demonstrated that CSCs are crucial for carcinogenesis, metastasis, tumor self-replication capacity, tumor metabolic reprogramming, and the remodeling of the tumor immunological microenvironment [24]. Factors highly correlated with tumor stemness were identified using a comprehensive bioinformatics analysis to obtain insights into the stemness features of PPGLs. A stemness scoring system was developed to predict patient prognosis and immunotherapy responsiveness.

The mRNAi is an index that describes the similarity between tumor and stem cells to explore their association with phenotypic characteristics. Now, PPGLs cannot be distinguished between benign and malignant. Instead, all PPGLs are considered potentially malignant [25]. Malignant metastasis occurs in 15% of pheochromocytomas and half of abdominal paragangliomas. Many studies also explore histological and molecular predictive markers to predict tumor metastasis [26]. Although current reports show that the probability of metastases in patients without *SDHB* mutations is low, some patients still develop metastasis [20,21]. Our study explored mRNAsi in patients without *SDHB* mutations and found that PPGLs patients with metastasis had higher mRNAsi scores. This suggests that the degree of tumor stemness of PPGLs affects the patient metastasis and progression, and it may predict the metastasis probability in patients without *SDHB* and cancer progression through changes in its stemness characteristics in the future.

The TME plays an important role in tumor cell renewal, proliferation, and differentiation, especially immune-related signals in the microenvironment that can affect patients’ prognosis and survival rate. According to previous studies, CSCs have a regulatory effect on tumor immunity, which is consistent with our findings in PCPG [27]. Our study revealed that mRNAsi scores were negatively correlated with immune, stromal, and ESTIMATE scores, suggesting that the stronger the stemness characteristics of PPGLs, the weaker their immune function, and the tumor may escape immune clearance under the condition of low immune function. Patients with a low mRNAsi risk exhibited high immune activation characteristics, and ultimately with a good clinical prognosis. As shown in Figure 4A, our study analyzed the infiltration characteristics of immune cells, between immune-activated (Immunity_H) and immune-suppressive (Immunity_L) subtypes, suggesting the TME landscapes in low mRNAsi and high mRNAsi risk patients. Notably, various immune cells that exert anti-tumor roles, such as M1-like macrophages, NK cells, and neutrophils, were enriched in patients with low mRNAsi risk. In addition, the infiltration of M2 macrophages with pro-tumor roles also significantly decreased than the high mRNAsi subtypes. Previous studies have reported that M1-like macrophages have an antitumorigenic role when the innate and acquired immune systems are activated. The dramatic infiltration of M1-like macrophages impaired the growth, migration, and angiogenesis of various solid tumors [28], which was related to a better survival rate. According to several studies, tumor-associated macrophages, particularly M2-like macrophages, directly communicate with CSCs to support their ability to migrate, self-renew, and survive. However, the effects of classical (M1)-polarized macrophages on CSCs are unclear. Luo et al. reported that unlike the alternative (M2)-polarized macrophages, M1-like macrophages inhibit the self-renewal and expansion of the hematopoietic stem cell (HSC) by inducing nitric oxide (NO) production from catalytic intracellular L-arginine, because the exposure to NO further induces HSC and hematopoietic progenitor cell apoptosis [29]. The above result also explains why PPGLs patients with low tumor stemness risk have a high proportion of M1-like macrophage infiltration. Many studies indicated that TAMs in the TME often exhibited M2 macrophages, characterized by the production and secretion of immunosuppressive cytokines. M2-like TAMs secreted cytokines, inhibited the function of cytotoxic T lymphocytes, blocked Th1-type immune activity, promoted anti-immunity Th2-type activity, thus allowing tumors to evade immune surveillance and thereby promoting tumor cell growth, drug resistance, and angiogenesis [30,31,32]. Therefore, compared with the high mRNAsi, the lower M2 macrophages in the low mRNAsi subtypes, also caused better clinical prognosis. Meanwhile, several immune checkpoint genes were suppressed in the high mRNAsi risk population, attenuating the immune response. Thus, we proposed that the CSC marker expression in tumor tissues of PPGLs has a close association with the immunosuppressed immune microenvironment and revealed poor survival outcomes.

Recently, immunotherapy has been widely investigated and applied to various tumors. Patient-tailored immunotherapy has provided tremendous advantages to patients with neural crest-derived cancer, especially for melanoma and neuroblastoma. However, the clinical scientific experience of these phase II clinical trials with checkpoint inhibitors and other types of immunotherapy for PPGLs was not satisfactory [33,34]. In the current study, PPGLs patients in the low-mRNAsi risk group had higher antitumor immune activity and immune checkpoint expression. We had a hunch that the mRNAsi risk value could reveal how much patients would benefit from immunotherapy. As expected, immunotherapy had a substantially better effect on patients classified as a low-risk group. IMvigor210, an authentic immune therapy cohort that investigated the efficacy of PD-L1 inhibitor therapy on patients with metastatic endothelial cancer, was used for validation. Our study demonstrated that the risk score model might be appropriate for predicting the efficacy of immunotherapy in PPGLs and beneficial for patients with other types of cancer. Medical staff can choose a different treatment strategy depending on the two different mRNAsi signature subtypes above. We believed that PPGLs patients with low miRNAsi risk, characterized by the immune-activated TME, could benefit more from immunotherapy, and in addition, their estimated IC50 for drugs such as erlotinib and I-BET-762 is lower. Some studies also indicated that the immunosuppressive tumor microenvironment (TME) has lower sensitivity to immunotherapy, for example, in prostate cancer [35]. Zhou et al. demonstrated the low-score stemness subgroups were the significant responders to anti-CTLA4/PD-1 treatment in clear cell renal cell carcinoma [36]. These findings were consistent with our results. Therefore, the response of tumor to treatment was also closely related to the interaction of TME. Many studies have found that neoadjuvant chemotherapy combined with immunotherapy could increase CD8+ T cell infiltration more significantly than neoadjuvant chemotherapy alone, prolonging overall patient survival [37]. Tumors with better treatment effects (higher pathological complete response) exhibited a high M1 polarization level before treatment, and appear to have a higher M1/M2 ratio after treatment [38,39].

Targeting tumor stem cells is the top priority in treating and preventing tumor recurrence. Bruno et al. proposed that disrupting CSC-TAM crosstalk or combining CSCs and TAMs targeting may be a new therapeutic strategy [40]. In the future, we can thoroughly grasp the mechanism of tumor stem cells and completely eliminate tumors at the stem level. Although we performed a prognostic analysis of our risk model in the TCGA dataset, we did not find other external sequencing datasets to validate it due to the rarity of PPGLs. We may discover relevant and complete follow-up data to supplement the experimental validation. Moreover, the mechanism of interaction between CSCs and various immune components needs further experimental exploration in the TME of PPGLs.

## 5. Conclusions

In conclusion, we explored the relationship between tumor stemness characteristics and the clinical properties and immune microenvironment of PPGLs in TCGA to develop a stemness scoring system that can be applied to predict prognosis and patient immunotherapy responsiveness. The occurrence, metastasis, and prognosis of malignancies are strongly correlated with the stemness characteristics of tumors. Therefore, evaluating tumor stemness may help determine the extent of the tumor and prognostic indicators and serve as a guide for future pharmacological target therapy.

## Data Availability

The original contributions presented in the study are included in the article/Supplementary Materials. Further inquiries can be directed to the corresponding authors.

## Supplementary Materials

The following supporting information can be downloaded at: https://www.mdpi.com/article/10.3390/bioengineering12030219/s1, Figure S1: Comparison of immune infiltration levels between high and low-risk patients by using (A) CIBERSORT, (B) EPIC, (C) xCell, (D) quantTIseq, (E) MCP-counter, (F) TIME, and (G) ssGSEA algorithms. * *p* < 0.05, ** *p* < 0.01; *** *p* < 0.001, ns, no statistical difference. Table S1: mRNAsi-associated prognostic genes.

## Abbreviations

PPGLs	Pheochromocytoma and paragangliomas
mRNAsi	mRNA expression-based stemness indices
TME	tumor microenvironment
WGCNA	weighted gene co-expression network analysis
CSCs	cancer stem cells
TCGA	The Cancer Genome Atlas
ssGSEA	single-sample Gene Set Enrichment Analysis
TIICs	tumor-infiltrating immune cells
KEGG	Kyoto Encyclopedia of Genes and Genomes
GO	The Gene Ontology

## References

[1] Calissendorff J., Juhlin C.C., Bancos I., Falhammar H. (2022). Pheochromocytomas and Abdominal Paragangliomas: A Practical Guidance. Cancers.

[2] Mete O., Asa S.L., Gill A.J., Kimura N., de Krijger R.R., Tischler A. (2022). Overview of the 2022 WHO Classification of Paragangliomas and Pheochromocytomas. Endocr. Pathol..

[3] Lenders J.W., Duh Q.Y., Eisenhofer G., Gimenez-Roqueplo A.P., Grebe S.K., Murad M.H., Naruse M., Pacak K., Young W.F., Endocrine S. (2014). Pheochromocytoma and paraganglioma: An endocrine society clinical practice guideline. J. Clin. Endocrinol. Metab..

[4] Chen H., Sippel R.S., O’Dorisio M.S., Vinik A.I., Lloyd R.V., Pacak K. (2010). The North American Neuroendocrine Tumor Society consensus guideline for the diagnosis and management of neuroendocrine tumors: Pheochromocytoma, paraganglioma, and medullary thyroid cancer. Pancreas.

[5] Gu H.Y., Qu W.Q., Peng H.H., Yu Y.F., Jiang Z.Z., Qi B.W., Yu A.X. (2022). Stemness Subtypes and Scoring System Predict Prognosis and Efficacy of Immunotherapy in Soft Tissue Sarcoma. Front. Immunol..

[6] Chen W., Hong Z., Kang S., Lv X., Song C. (2022). Analysis of Stemness and Prognosis of Subtypes in Breast Cancer Using the Transcriptome Sequencing Data. J. Oncol..

[7] Yuan H., Yu Q., Pang J., Chen Y., Sheng M., Tang W. (2022). The Value of the Stemness Index in Ovarian Cancer Prognosis. Genes.

[8] Fishbein L., Leshchiner I., Walter V., Danilova L., Robertson A.G., Johnson A.R., Lichtenberg T.M., Murray B.A., Ghayee H.K., Else T. (2017). Comprehensive Molecular Characterization of Pheochromocytoma and Paraganglioma. Cancer Cell.

[9] Malta T.M., Sokolov A., Gentles A.J., Burzykowski T., Poisson L., Weinstein J.N., Kamińska B., Huelsken J., Omberg L., Gevaert O. (2018). Machine Learning Identifies Stemness Features Associated with Oncogenic Dedifferentiation. Cell.

[10] Cheng J., Yang L., Kumar V., Agarwal P. (2014). Systematic evaluation of connectivity map for disease indications. Genome Med..

[11] Newman A.M., Liu C.L., Green M.R., Gentles A.J., Feng W., Xu Y., Hoang C.D., Diehn M., Alizadeh A.A. (2015). Robust enumeration of cell subsets from tissue expression profiles. Nat. Methods.

[12] Racle J., Gfeller D. (2020). EPIC: A Tool to Estimate the Proportions of Different Cell Types from Bulk Gene Expression Data. Methods Mol. Biol..

[13] Aran D., Hu Z., Butte A.J. (2017). xCell: Digitally portraying the tissue cellular heterogeneity landscape. Genome Biol..

[14] Finotello F., Mayer C., Plattner C., Laschober G., Rieder D., Hackl H., Krogsdam A., Loncova Z., Posch W., Wilflingseder D. (2019). Molecular and pharmacological modulators of the tumor immune contexture revealed by deconvolution of RNA-seq data. Genome Med..

[15] Becht E., Giraldo N.A., Lacroix L., Buttard B., Elarouci N., Petitprez F., Selves J., Laurent-Puig P., Sautes-Fridman C., Fridman W.H. (2016). Estimating the population abundance of tissue-infiltrating immune and stromal cell populations using gene expression. Genome Biol..

[16] Li T., Fan J., Wang B., Traugh N., Chen Q., Liu J.S., Li B., Liu X.S. (2017). TIMER: A Web Server for Comprehensive Analysis of Tumor-Infiltrating Immune Cells. Cancer Res..

[17] Xu L., Deng C., Pang B., Zhang X., Liu W., Liao G., Yuan H., Cheng P., Li F., Long Z. (2018). TIP: A Web Server for Resolving Tumor Immunophenotype Profiling. Cancer Res..

[18] Jiang P., Gu S., Pan D., Fu J., Sahu A., Hu X., Li Z., Traugh N., Bu X., Li B. (2018). Signatures of T cell dysfunction and exclusion predict cancer immunotherapy response. Nat. Med..

[19] Mariathasan S., Turley S.J., Nickles D., Castiglioni A., Yuen K., Wang Y., Kadel E.E., Koeppen H., Astarita J.L., Cubas R. (2018). TGFbeta attenuates tumour response to PD-L1 blockade by contributing to exclusion of T cells. Nature.

[20] Perez K., Jacene H., Hornick J.L., Ma C., Vaz N., Brais L.K., Alexander H., Baddoo W., Astone K., Esplin E.D. (2022). SDHx mutations and temozolomide in malignant pheochromocytoma and paraganglioma. Endocr. Relat. Cancer.

[21] Hescot S., Curras-Freixes M., Deutschbein T., van Berkel A., Vezzosi D., Amar L., de la Fouchardiere C., Valdes N., Riccardi F., Do Cao C. (2019). Prognosis of Malignant Pheochromocytoma and Paraganglioma (MAPP-Prono Study): A European Network for the Study of Adrenal Tumors Retrospective Study. J. Clin. Endocrinol. Metab..

[22] Marquardt S., Solanki M., Spitschak A., Vera J., Putzer B.M. (2018). Emerging functional markers for cancer stem cell-based therapies: Understanding signaling networks for targeting metastasis. Semin. Cancer Biol..

[23] Najafi M., Farhood B., Mortezaee K. (2019). Cancer stem cells (CSCs) in cancer progression and therapy. J. Cell. Physiol..

[24] La Noce M., Paino F., Mele L., Papaccio G., Regad T., Lombardi A., Papaccio F., Desiderio V., Tirino V. (2018). HDAC2 depletion promotes osteosarcoma’s stemness both in vitro and in vivo: A study on a putative new target for CSCs directed therapy. J. Exp. Clin. Cancer Res..

[25] Juhlin C.C. (2021). Challenges in Paragangliomas and Pheochromocytomas: From Histology to Molecular Immunohistochemistry. Endocr. Pathol..

[26] Granberg D., Juhlin C.C., Falhammar H. (2021). Metastatic Pheochromocytomas and Abdominal Paragangliomas. J. Clin. Endocrinol. Metab..

[27] Ren B., Cui M., Yang G., Wang H., Feng M., You L., Zhao Y. (2018). Tumor microenvironment participates in metastasis of pancreatic cancer. Mol. Cancer.

[28] Najafi M., Hashemi Goradel N., Farhood B., Salehi E., Nashtaei M.S., Khanlarkhani N., Khezri Z., Majidpoor J., Abouzaripour M., Habibi M. (2019). Macrophage polarity in cancer: A review. J. Cell. Biochem..

[29] Luo Y., Shao L., Chang J., Feng W., Liu Y.L., Cottler-Fox M.H., Emanuel P.D., Hauer-Jensen M., Bernstein I.D., Liu L. (2018). M1 and M2 macrophages differentially regulate hematopoietic stem cell self-renewal and ex vivo expansion. Blood Adv..

[30] Singer M., Zhang Z., Dayyani F., Zhang Z., Yaghmai V., Choi A., Valerin J., Imagawa D., Abi-Jaoudeh N. (2024). Modulation of Tumor-Associated Macrophages to Overcome Immune Suppression in the Hepatocellular Carcinoma Microenvironment. Cancers.

[31] Xiang X., Wang J., Lu D., Xu X. (2021). Targeting tumor-associated macrophages to synergize tumor immunotherapy. Signal Transduct. Target. Ther..

[32] Moeini P., Niedźwiedzka-Rystwej P. (2021). Tumor-Associated Macrophages: Combination of Therapies, the Approach to Improve Cancer Treatment. Int. J. Mol. Sci..

[33] Jimenez C., Armaiz-Pena G., Dahia P.L.M., Lu Y., Toledo R.A., Varghese J., Habra M.A. (2022). Endocrine and Neuroendocrine Tumors Special Issue-Checkpoint Inhibitors for Adrenocortical Carcinoma and Metastatic Pheochromocytoma and Paraganglioma: Do They Work?. Cancers.

[34] Fanciulli G., Di Molfetta S., Dotto A., Florio T., Feola T., Rubino M., de Cicco F., Colao A., Faggiano A., Nike G. (2020). Emerging Therapies in Pheochromocytoma and Paraganglioma: Immune Checkpoint Inhibitors in the Starting Blocks. J. Clin. Med..

[35] Zheng K., Hai Y., Xi Y., Zhang Y., Liu Z., Chen W., Hu X., Zou X., Hao J. (2023). Integrative multi-omics analysis unveils stemness-associated molecular subtypes in prostate cancer and pan-cancer: Prognostic and therapeutic significance. J. Transl. Med..

[36] Zhou P., Hu H., Lu Y., Xiao J., Wang Y., Xun Y., Xu J., Liu C., Wang S., Hu J. (2022). Cancer stem/progenitor signatures refine the classification of clear cell renal cell carcinoma with stratified prognosis and decreased immunotherapy efficacy. Mol. Ther. Oncolytics.

[37] Qiu Z., Li Z., Liu X., Zhang R., Li Y., Gao C., Mao X., Bao Y., Zhang M., Guo C. (2024). From tumor microenvironment to emerging biomarkers: The reshaping of the esophageal squamous cell carcinoma tumor microenvironment by neoadjuvant chemotherapy combined with immunotherapy. Front. Immunol..

[38] Liu Z., Zhang Y., Ma N., Yang Y., Ma Y., Wang F., Wang Y., Wei J., Chen H., Tartarone A. (2023). Progenitor-like exhausted SPRY1^+^CD8^+^ T cells potentiate responsiveness to neoadjuvant PD-1 blockade in esophageal squamous cell carcinoma. Cancer Cell.

[39] Wang S., Xu G., Li M., Zheng J., Wang Y., Feng X., Luo J., Wang S., Liu H., Duan W. (2023). M1 macrophage predicted efficacy of neoadjuvant camrelizumab combined with chemotherapy vs chemotherapy alone for locally advanced ESCC: A pilot study. Front. Oncol..

[40] Sainz B., Carron E., Vallespinos M., Machado H.L. (2016). Cancer Stem Cells and Macrophages: Implications in Tumor Biology and Therapeutic Strategies. Mediat. Inflamm..

