# Supplementary material for: Molecular Signatures of Cancer Stemness Characterize the Correlations with Prognosis and Immune Landscape and Predict Risk Stratification in Pheochromocytomas and Paragangliomas
Source: Bioengineering (Basel). 2025 Feb 21;12(3):219. doi: 10.3390/bioengineering12030219 (PMC11939611; doi:10.3390/bioengineering12030219)
Supplement: Supplementary file 1 [file bioengineering-12-00219-s001.zip › bioengineering-3413881-supplementary-english.pdf]

Supplementary Table S1. mRNAsi-associated prognostic genes

Gene	p-value
MYO3A	0.04293056
C4orf31	0.033382732
LOC284276	0.023615622
CCL26	0.026113765
EBF2	0.038293477
DACT2	0.042961987
LOC400804	0.030484662
LOC401463	0.03086345
WDR16	0.007423053
LOC285954	0.043014128
TPSD1	0.023991055
FRRS1	0.041050968
C10orf105	0.044693341
GPR148	0.028368266
TBX1	0.004710123
CLCNKB	0.042229258
PATE2	0.02128643
FAM3B	0.007043236
C15orf54	0.029369045
FLG2	0.047620996
GPR88	0.034971363
MDS2	0.027330854

MUC15	0.009405067
TMEM155	0.022416983
PZP	0.03866282
GATA4	0.002771508
SOX11	0.041628055
LOC100188947	0.040343334
LY6H	0.003293685
C1orf94	0.018558682
CRYBB3	0.028774623
TREM1	0.021195239
FGF22	0.007636695
